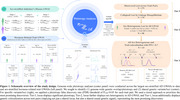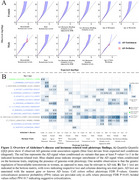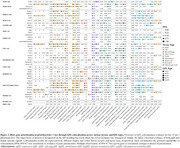# Genetic Pleiotropy Across Hormone‐Related Traits and Alzheimer's Disease Reveals Sex‐Specific Relationships and Risk Genes

**DOI:** 10.1002/alz70855_103719

**Published:** 2025-12-24

**Authors:** Chenyu Yang, Noah Cook, Danielle M. Reid, Michael E. Belloy

**Affiliations:** ^1^ NeuroGenomics and Informatics Center, Washington University School of Medicine, St Louis, MO, USA; ^2^ Department of Neurology, Washington University School of Medicine, St Louis, MO, USA

## Abstract

**Background:**

Sex differences are pervasive in Alzheimer's disease (AD). While there is evidence for sex‐specific genetic risk and the influence of hormonal factors in AD, their interplay is not well understood. In this study, we performed pleiotropy analyses between genome‐wide association studies (GWASs) on hormone‐related traits and AD, aiming to provide new insights into sex‐specific mechanisms underlying AD.

**Method:**

**Figure 1** summarizes the study design for pleiotropy analyses across hormone‐related GWASs. We used both sex‐matched analyses and a comparison of sex‐stratified hormone‐related GWASs with non‐stratified AD GWASs, anticipating the latter provides additional power when sex‐biased AD genetic signals reside at subthreshold levels and might otherwise be. We then sought to identify (1) genome‐wide genetic overlap/pleiotropy and (2) shared genetic variants/loci. We implemented a tiered approach to prioritize the most promising discoveries, using genetic colocalization analyses to identify loci with shared causal variants across respective hormone‐AD trait‐pairs. Tier 1 loci were subjected to additional quantitative trait locus (QTL) colocalization analyses to prioritize likely causal genes.

**Result:**

For both men and women, we observed genome‐wide pleiotropy enrichment of AD genetic signals when conditioned on any hormone‐related trait, except age‐at‐voice‐breaking and bioavailable testosterone in men (Figure 2A). Particularly, genetic regulation of bioavailable testosterone showed AD enrichment in women. We identified 178 independent pleiotropic loci, 15 of which were prioritized as tier 1 loci, with 11 out of the 15 not previously linked to AD (Figure 2B). Sex‐specificity was balanced, with 8 out of 15 tier 1 loci being female‐biased. Follow‐up QTL colocalization prioritized risk genes for 12 out of 15 tier 1 loci (Figure 3). Notably, at an AD signal in men at the *CEPNW* locus, *NCOA7* was prioritized, which encodes an Estrogen Receptor‐Associated Protein linked to developmental disorders that also regulates vacuolar ATPase and lysosomal activity, suggesting its connection to AD pathogenesis. Prioritization of *FADS1* and *FADS2* at the *FADS2* locus suggests that regulation of fatty acid metabolism may contribute to female‐specific AD risk.

**Conclusion:**

This study highlights sex‐specific genetic links between AD and hormone‐related traits, prioritizing 15 compelling loci for further investigation. These findings provide an inroad to elucidate sex‐specific AD pathways and drug targets.